# Training on handover of patient care within UK medical schools

**DOI:** 10.3402/meo.v18i0.20169

**Published:** 2013-01-11

**Authors:** Morris Gordon

**Affiliations:** 1 Department of Midwifery, Faculty of Health and Social care, University of Salford, Salford, UK; 2 Department of Paediatrics, Blackpool Victoria Hospital, Blackpool, UK

**Keywords:** handover, handoff, patient safety, non-technical skills, undergraduate medical education

## Abstract

**Background:**

Much evidence exists to demonstrate that poor handover can directly impact patient safety. There have been calls for formal education on handover, but evidence to guide intervention design and implementation is limited. It is unclear how undergraduate medical schools are tackling this issue and what barrier or facilitators exist to handover education. We set out to determine curriculum objectives, teaching and assessment methods, as well as institutional attitudes towards handover within UK medical schools.

**Methods:**

A descriptive, non-experimental, cross-sectional study design was used. A locally developed online questionnaire survey was sent to all UK Medical Schools, after piloting. Descriptive statistics were calculated for closed-ended responses, and free text responses were analysed using a grounded theory approach, with constant comparison taking place through several stages of analysis.

**Results:**

Fifty percent of UK medical schools took part in the study. Nine schools (56%) reported having curriculum outcomes for handover. Significant variations in the teaching and assessments employed were found. Qualitative analysis yielded four key themes: the importance of handover as an education issue, when to educate on handover, the need for further provision of teaching and the need for validated assessment tools to support handover education.

**Conclusions:**

Whilst undergraduate medical schools recognised handover as an important education issue, they do not feel they should have the ultimate responsibility for training in this area and as such are responding in varying ways. Undergraduate medical educators should seek to reach consensus as to the extent of provision they will offer. Weaknesses in the literature regarding how to design such education have exacerbated the problem, but the contemporaneous and growing published evidence base should be employed by educators to address this issue.

## Background

Handover or handoff can be defined as the passing of responsibility and information, both in varying quantities, between shifts or locations. Handover has been identified as a vulnerable period in the care process during which information may be lost, distorted, or misinterpreted (1–3), and this can directly impact patient safety (4, 5). Recent global moves to reduce working hours amongst medical staff, along with reconfigurations in services have increased the frequency and complexity of handover (6). There is much published work discussing ways to improve handover, mostly focussing on systems to manage information, such as standardised proformas (7, 8) or electronic handover systems (9, 10), although there is a corresponding paucity of evidence as to their effectiveness (6).

There have been calls for formal education on handover (11) and work has started to clarify competencies for training (12). In addition, handover is increasingly being recognised within graduate curriculum, with examples in the United Kingdom (13) and the United States (14). The published research on handover has tended to not be highly concerned with education (15), with only 10% of handover improvement projects being categorised as involving teaching or training. This author recently completed a systematic review of educational interventions to improve handover (16) that found a paucity of research investigating this issue, although this field is growing rapidly. Limited evidence was found to demonstrate that skills could be transferred into the workplace and no evidence was found that could improve patient outcomes. More importantly and as is often the case with evidence synthesis in medical education, a lack of published work describing the theoretical underpinning or pedagogical foundations of interventions was discovered. Educators are left with the problem of enhancing provision with limited evidence to guide on how to do so, even if evidence suggests that such education can be effective.

Given this lack of evidence and the clear need for handover education in some form, undergraduate medical education institutions are also being expected to train and assess elements of handover of care. However, the current state of this training within medical schools, the type of education being offered and how assessments are being made, remain unclear.

We set out to determine the current state of handover training within undergraduate medical schools in the United Kingdom and institutional attitudes to identify any common facilitators or barriers to handover education.

## Methods

A descriptive, non-experimental, cross-sectional study design was used. An online questionnaire survey was employed. The Questionnaire was developed locally for this study and piloted before its delivery by email using the online service ‘Surveymonkey’. It consisted of mainly closed questions, with some open-ended questions to gather qualitative data. Key educational personnel within each school in the United Kingdom were contacted and a single respondent was identified to take part. Therefore, the whole population sample of UK medical schools was invited to participate.

Descriptive statistics for closed-ended responses were compiled and analysed. Free text responses were analysed using a grounded theory approach (17). Anonymous responses were compiled and coded for key items. The analysis proceeded through three stages, consisting of open, axial and selective coding, with constant comparisons taking place throughout each phase (18). Each stage provided categories that could be used to explore the themes of the data and further inform the next stage of analysis.

## Results

### Response rate

A total of 19 out of the 32 UK medical schools invited to participate responded (14 from England, three from Scotland and one each from Northern Ireland and Wales). Of these, three schools declined to take part, with two reporting that school policy dictated they could not complete such studies and one school asking for local ethical approval. An ethics application was made, but no response was received at 12 weeks and so this was abandoned. This left a sample of 16 (50%) UK medical schools, with each country in the United Kingdom represented.

### Curriculum

Nine schools (56%) reported having curriculum aims, objectives or outcomes regarding the ability for graduates to handover, whilst the remaining schools had none. It was reported that handover was addressed from semester 1 in one school, but within the final semester of the course in the remaining schools. Several respondents mentioned patient safety as the driver for including handover in the curriculum.

### Teaching and assessment methods

As half of the schools did not recognise handover within their curriculum, there was no provision. Amongst the remaining schools, there was considerable variation in methods. This has been summarised in Table 1.

**Table 1:** Teaching and assessment methods reported amongst the 16 undergraduate medical schools studied

Teaching methods	No. of institutions	Assessment	No. of institutions
Observation during training	16	Objective structured clinical exam	5
Communication skill courses	6	Ward-based direct assessment	4
Case-based discussions	5	Communication skills exam	2
Reflective exercises	3	Reflective exercises	1
Lectures	3	Written assignments	1
e-Learning	3	Online assessment	1
Problem-based learning	2	Review of logbook	1
Ward simulation exercise	1	Ward simulation exercise	1

### Institutional view on handover education

Thirteen schools (81%) felt that handover needs specific training and that it is an important educational issue. Fourteen schools (88%) agreed that they would like to see more published educational research on handover. However, 81% did not agree that handover is an important issue for undergraduate education.

### Free text responses and themes

**Fig. 1:**
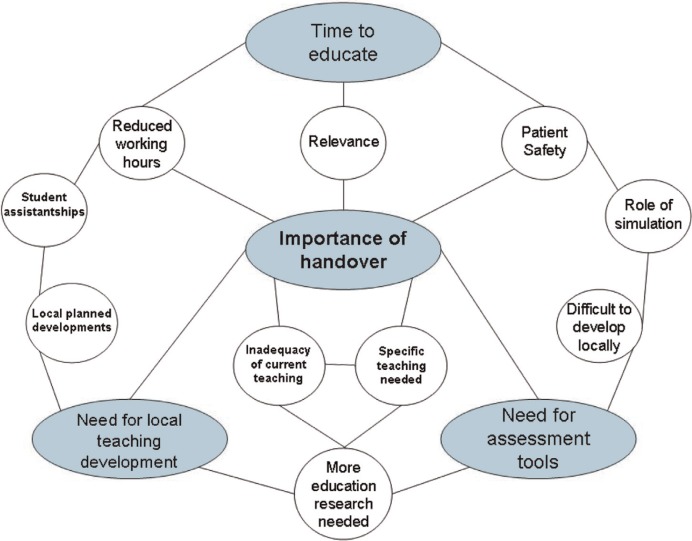
Map of key themes at the axial level of data analysis.

There were 223 items recorded in the open phase of coding. As analysis proceeded through the axial phase of coding, a number of themes were synthesised into a theme map (Fig. 1). At the selective level of analysis, this led to the four key themes below:

1. *Handover as a key educational issue:* Responders overwhelmingly agreed that handover is an increasingly important issue, identifying the drivers already mentioned. In particular, patient safety was the unifying area of alignment.
2. *When to educate on handover:* The majority of institutions felt that handover should be an educational objective for early graduate or ‘on the job’ training and that it did not sit well in a busy undergraduate programme.
3. *Need for further development of teaching:* Despite the views above, most schools felt they should be developing more interventions and were doing so.
4. *Requirement for formal assessment tools:* A lack of validated assessment tools was a key concern expressed, although most schools were currently assessing handover through existing methods.

## Discussion

This is the first study to examine education in the area of handover over a large sample of institutions. The key finding is ambivalence amongst UK undergraduate schools regarding this issue. They strongly agree that handover education is important and are compelled to develop teaching in this area, but they also strongly expressed the view that this is an issue that should be dealt with within postgraduate training. As a result of these conflicting views, schools are responding in varying ways, with significant difference in the provision being offered. Half of the schools are essentially not addressing handover education at present. The other half is using a range of teaching and assessment methods, again with no consensus. It is worth noting that no institution reported alignment with any conceptual frameworks or theoretical models when discussing handover training and this is probably the only unifying finding of this study. This most probably reflects weaknesses in the literature already identified, but clearly is a concern as the effectiveness of any provision made will be impacted by this lack of appropriate theoretical alignment or underpinning.

The view that handover education should occur in the postgraduate training is at odds with an identified model (16), which views handover not as a free standing issue, but built on expertise in a range of generic skills (16). These three overlapping areas are: (1) information transfer and systems of managing information; (2) responsibility and accountability; (3) elements in place to facilitate handover within the healthcare environment, such as teamwork and leadership. This skill set frames handover education as both a technical and non-technical skill (19). As such, these skills should be acquired from the very start of undergraduate training. It may be appropriate to address the specific issue of handover information management systems within the postgraduate setting, but skills in team working, communication and professionalism are key areas that should be addressed before graduation, both in the context of handover education, as well as part of the generic non-technical skill set all graduates require. Work using the handover educational model in this way (20) to design undergraduate teaching has begun and suggests its application is appropriate and pedagogically sound.

Therefore, the key barrier to development of handover education does not seem to be the lack of literature or evidence on the issue, but a lack of consensus amongst undergraduate medical institutions as to the extent of provision they must offer. Whilst it is outside of the scope of this work to suggest what form that provision should take, it is clear that the lack of consensus is impacting students, who almost certainly do not have a uniform set of skills. It seems reasonable to suspect this problem is not unique to the United Kingdom and is likely to reflect a global issue surrounding a relatively new issue in medical education.

The current concern regarding a lack of formalised and validated tools for handover education is a valid one and must be addressed. Clearly, this is difficult as there is not an even consensus regarding competencies in this area (12), although recently the first tool for assessing handover has been reported in the literature (21). This tool is based on the mini-clinical encounter exercise work based assessment and whilst not formally validated, offers an interesting development to educators.

In addition, the issue of effectiveness of developments in handover education must also be considered. Even though this is the focus of most existing literature, it has been poorly answered. This outcome is limited to those demonstrating changes in attitudes or knowledge and skills, with minimal demonstrating changes in behaviour. Whilst the goal of handover education is clearly focussed on improving patient safety, there is no evidence that handover education, evidence based or otherwise, is able to actually improve the safety of patients (22). Any future work aimed at designing, implementing and assessing undergraduate handover education must attempt to address this issue.

There are some key limitations to these findings that must be considered, mostly regarding risk of bias. This study was based in the United Kingdom only and whilst a large sample was included, there is the possibility of a bias amongst interested respondents. In addition, acceptability bias amongst respondents may also limit the usefulness of some of these findings. Finally, the qualitative data analysis could be influenced by the single author's views and personal biases.

## Conclusions

Whilst undergraduate medical schools recognised handover as an important education issue, they do not feel that they should have the ultimate responsibility for training in this area and as such are responding in varying ways. Undergraduate medical educators should seek to reach consensus as to the extent of provision they will offer. Weaknesses in the literature regarding how to design such education have exacerbated the problem, but the contemporaneous and growing published evidence base should be employed by educators to address this issue.
